# Myopia as a risk factor for subsequent retinal tears in the course of a symptomatic posterior vitreous detachment

**DOI:** 10.1186/s12886-017-0629-6

**Published:** 2017-12-01

**Authors:** Nicolas Crim, Evangelina Esposito, Rodolfo Monti, Leandro J. Correa, Horacio M. Serra, Julio A. Urrets-Zavalia

**Affiliations:** 1 Department of Ophthalmology, University Clinic Reina Fabiola, Catholic University of Cordoba, Oncativo 1248, 5000 Cordoba, Argentina; 2 CIBICI, Faculty of Chemical Sciences, National University of Cordoba, Haya de la Torre esq. Medina Allende, Ciudad Universitaria, 5000 Cordoba, Argentina

**Keywords:** Retinal tear, Posterior vitreous detachment, Retinal detachment, Myopia

## Abstract

**Background:**

Retinal tears complicating the course of a posterior vitreous detachment (PVD) may be unique or multiple, and when multiple they may occur simultaneously or subsequently at different moments in the evolution of a PVD. The purpose of our study was to analyze the prevalence of subsequent retinal tears (SRT) in patients with a PVD, and to identify possible risk factors for SRT.

**Methods:**

One hundred and seventy six eyes in 165 consecutive patients that presented one or more retinal tears in the evolution of a symptomatic PVD, with a minimum follow-up of 12 months, were retrospectively evaluated. The primary outcome measure was to characterize the clinical features associated with SRT formation against those eyes with non-subsequent retinal tear (NSRT-retinal tear/s diagnosed at initial examination) formation. For that purpose, this cohort of patients was divided into two different groups: *group 1* included eyes presenting one or multiple retinal tears only at initial examination (NSRT), and *group 2* eyes that progressed to a further retinal tear/s (SRT) during follow-up.

**Results:**

Group 1 comprised 154 eyes from 145 patients, 48.7% males and 51.3% females with a mean age of 56.9 ± 14.0 years (range = 15-89); 17.2% of patients had a previous retinal tear or retinal detachment in the fellow eye; mean number of retinal tears per eye 1.42 ± 0.8 (range = 1-5); 20.8% presented bilateral retinal tears; 59.1% were myopic eyes (*p* < 0.05). Group 2 comprised 22 eyes from 20 patients; mean age was 53.3 ± 13.6 years (range = 30-69); 63.6% were male (*p* = 0.13), and 7 patients (31.8%) had a history of SRT or retinal detachment in the fellow eye (p = 0.13). The mean number of retinal tears per eye was 1.36 ± 0.5 (range = 1-2); bilateral retinal tears were noted in 18.2% of eyes; 86.4% were myopic eyes (*p* = 0.01); 81.8% occurred within a 120 days-period following diagnosis of the first retinal tear.

**Conclusions:**

Multiple retinal tears may be diagnosed in the evolution of a PVD. SRT are most frequently observed in myopic patients, and are usually symptomatic. Follow-up must extend for at least 4 months after the initial symptoms.

## Background

Posterior vitreous detachment (PVD) represents most frequently the end-stage of an age-related progressive degenerative process of the vitreous body [1]. PVD may also occur early in life under certain pathological circumstances such as high myopia, heredo-degenerative diseases of the retina, certain vitreoretinopathies, uveitis, trauma, and vitreous hemorrhage [2–4].

The vitreous base constitutes a circumferential band-shaped site of most firm adhesion of the vitreous body to the ciliary non-pigmented epithelium and the retro-oral underlying retina [1, 5]. Its width increases progressively with age, extending more posteriorly in nasal quadrants than in temporal quadrants, as observed by Wang and coworkers in 58 pairs of human autopsy eyes [5]. A smooth posterior border of the vitreous base depends on the pattern of collagen linkage across the internal limiting lamina [5]. Anomalous posterior extension of the vitreous base and anomalies in its posterior border configuration may constitute risk factors for the development of a retinal tear/s as the consequence of vitreoretinal tractions exerted in the course of a PVD [5–7]. Retinal tears have been found in 14.3% of autopsy eyes with a PVD [7], and clinically in 8% to 15% of patients presenting a PVD [8–12].

Retinal tears complicating the course of a PVD may be unique or multiple, and when multiple they may occur simultaneously or subsequently at different moments in the evolution of a PVD [4, 13]. Delayed retinal tears may develop during the evolution of an incomplete PVD persisting for a long time [14], and persistent vitreo-retinal traction on symptomatic retinal tear/s could be complicated by a retinal detachment in 30% to 50% of cases [15–18].

The purpose of our study was to analyze the prevalence of subsequent retinal tears (SRT) in patients that developed retinal tears in the course of a PVD and to identify possible predisposing risk factors for their occurrence.

## Methods

One hundred and seventy-six eyes in 158 consecutive patients that presented one or more retinal tears in the evolution of a PVD, evaluated in the department of ophthalmology at a university hospital and followed for at least 12 months, were retrospectively analyzed and eligible for inclusion. Patients with previous retinal detachment in the presenting eye, a history of uveitis, or ocular trauma, were excluded from the study, as were eyes that had any media opacification (corneal scar, cataract, vitreous hemorrhage) that precluded adequate fundus examination.

Besides a comprehensive ophthalmological examination, fundus biomicroscopy and indirect ophthalmoscopy with scleral depression were performed in all 158 patients by the same examiner (JAUZ). Diagnosis of PVD was made on the basis of the presence of a mobile Weiss ring and/or visualization of an undulating posterior hyaloid by direct slit-lamp biomicroscopy of the vitreous cavity, or on fundus biomicroscopy by means of a 3-mirror lens or a + 78 non-contact lens.

This cohort of patients was divided into two different groups: *group 1* included eyes presenting one or multiple retinal tears only at initial examination (NSRT), and *group 2* included eyes that progressed subsequently to a further retinal tear/s (SRT) observed days or weeks during follow-up.

An eye was considered emmetrope when cycloplegic refraction was between −0.50 and +0.50 and axial length between 22 and 24 mm, hyperopic when cycloplegic refraction was over +0.50 and axial length of less than 22 mm, and myopic when cycloplegic refraction was over −0.50 and axial length of more than 24 mm.

Symptoms were registered as floaters alone, floaters with flashes, flashes alone, and visual acuity loss.

Visual acuity was recorded with a Snellen chart and converted into LogMAR units.

A retinal tear was considered of small size if it measured less than 1 disc diameter (DD) in its major longest axis, medium size between 1 and 2 DD, or large size if larger than 2 DD.

According to retinal topographic peripheral distribution, retinal tears were grouped as to be located superotemporal, inferotemporal, superonasal, and inferonasal.

The primary outcome measure was to characterize the clinical features associated with SRT against those eyes with NSRT formation.

The study was approved by the institutional review board of the University Clinic Reina Fabiola from the Catholic University of Cordoba, and conducted in accordance with the tenets of the Declaration of Helsinki. An informed consent was obtained from all patients participating in the study.

Statistical analysis was performed by means of the Student’s t test or the Mann-Whitney test. Logistic regression and Spearman correlation were performed for categorized data. Chi square and odds ratio were used to determine risk factor. InfoStat was the statistical software used. Statistical significance was considered when *p* < 0.05.

## Results

### Group 1

(NSRT) conprised 154 (87.5%) eyes from 145 patients that met the inclusion criteria, namely having one or more retinal tears at presentation but no further tear formation. The mean age at the moment of diagnosis was 56.9 ± 14.0 years (range = 15-87), 48.7% being males and 51.3% females. A previous retinal tear and/or retinal detachment in the fellow eye was noted in 17.2% of patients, while the mean number of retinal tears per eye was 1.39 ± 0.8 (range = 1-5). Bilateral retinal tears were found at presentation in 22.1% of patients, and 59.1% of the eyes were myopic (Tables 1 and 2).

**Table 1:** Demography and clinical data of patients and eyes from groups 1 (non-subsequent retinal tears) and 2 (subsequent retinal tears)

		Group 1	Group 2	*P* values
		NSRT	SRT	
Patients		145 (87.5%)	20 (12.5%)	
Eyes		154 (87.5%)	22 (12.5%)	–
Patients with bilateral retinal tear/s or retinal detachment in fellow eye		26 (17.2%)	7 (31.8%)	0.10
Age of patients		56.9 ± 14	53.3 ± 13.6	0.37
		(*r* = 15-89)	(*r* = 30-69)	
Gender	Female	79 (51.3%)	8 (36.4%)	0.37
	Male	75 (48.7%)	14 (63.6%)	

*NSRT* non-subsequent retinal tears, *SRT* subsequent retinal tears, *PVD* posterior vitreous detachment, *r* range

**Table 2:** Results per group according to symptoms, pseudophakic status, complications after treatment of tear/s, occurrence of retinal detachment, and myopia

	Group 1	Group 2	*P* values
	NSRT	SRT	
	n (%)	n (%)	
Symptoms			
Floaters	108 (70.1%)	17 (77.3%)	>0.05
Floaters with flashes	29 (18.8%)	2 (9.1%)	
Flashes	10 (6.5%)	1 (4.5%)	
Visual acuity loss	7 (4.5%)	2 (9.1%)	
Pseudophakia	19 (12.3%)	2 (9.1%)	0.66
Complications after treatment	15 (9.7%)	8 (36.4%)	<0.001*
Myopic eyes	91 (59.1%)	19 (86.4%)	0.01

* indicates statistical significance

### Group 2

(SRT) comprised 22 (12.5%) eyes from 20 patients with a mean age of 53.3 ± 13.6 years (range = 30-69), males being more prevalent (63.6%); 31.8% of patients had a previous retinal tear or retinal detachment in the fellow eye. 86.4% of these eyes were myopic. SRT occur within a period of 60 days after the initial finding of a retinal tear in 72.7% of eyes, and in the 120 days period that followed the initial diagnosis of a tear in 81.8% of eyes (Tables 1 and 2).

Concerning symptoms, no statistical significance between NSRT and SRT patients was found; however, floaters were more frequent in both groups at initial presentation (70.1% and 77.27% respectively).

Myopia was more frequently observed among patients presenting SRT (86.4% vs. 59.1% in NSRT) (*p* = 0.01; adjusted OR 4.38; 95% CI, 1.34 to 14.30). SRT eyes had longer axial length than NSRT (medians = 24.5 mm and 26.34 mm, respectively; *p* = 0.001).

After an initial laser treatment for a retinal tear/s, some patients developed a retinal detachment or a vitreous hemorrhage. These complications were more prevalent in SRT group than in NSRT (36.4% (8) vs. 9.7% (15), respectively; adjusted OR, 5.30; 95% CI, 1.96 to 14.34, *p* < 0.001).

Visual acuity did not show differences between NSRT and SRT in the initial examination, being medians 0.20 and 0.40 LogMAR respectively (*p* = 0.22). However, initial visual acuity was lower in cases with a history of retinal detachment o retinal tear/s in the fellow eye.

An inverse relationship between initial visual acuity and axial length was observed in both groups, where lower visual acuities were observed among eyes with higher axial lengths (*p* = 0.01).

Small retinal tears were more prevalent in both groups, but no significant differences concerning size, number, or location of retinal tear, were found between NSRT and SRT (Table 3).

**Table 3:** Retinal tears characteristics (number, location of initial tear/s, size)

	Group 1	Group 2	P values
	NSRT	SRT	
Number of retinal tears per eye	1.42 ± 0.8 (*r* = 1-5)	1.36 ± 0.5 (*r* = 1-2)	0.54
	n (%)	n (%)	
Initial retinal tear location			
Superotemporal	80 (45.5)	11 (40.7)	0.94
Inferotemporal	53 (30.1)	12 (44.5)	0.21
Superonasal	29 (16.5)	2 (7.4)	0.32
Inferonasal	14 (7.9)	2 (7.4)	0.92
Retinal tear size			
Small	108 (66.3)	11 (47.8)	0.27
Medium	44 (27)	9 (39.1)	0.45
Large	11 (6.7)	3 (13.1)	0.32

Retinal detachment during follow up was observed more frequently in eyes with SRT (8 eyes-36%) than in eyes with NSRT (16 eyes-8%) (OR 6.39; CI 95%, 2.39-17.13; *p* = 0.001).

In the SRT group, 18 eyes (81.8%) were found to have a delayed retinal tear during the four-month period following the initial presentation, whereas in 20 (90.9%) of the 22 eyes delayed tears were detected within the 12-month follow-up period from presentation. Only in 2 eyes the interval was longer than 12 months from presentation. These two eyes were both highly myopic and both had a history of a retinal detachment in the fellow eye.

## Discussion

Retinal tears constitute the most frequent and potentially hazardous complication of a PVD [10, 12], being symptomatic in a significant majority of cases [19–21].

In our series, SRT occurred in 12.5% of eyes with retinal tears complicating a PVD. These figures are similar to those reported by Sharma et al. [13], who observed 19 SRT in 204 eyes with retinal tears (9.3%), with a mean follow-up of 16.6 months (*r* = 1-157 months).

Bilateral tears were more frequently observed in patients with SRT (31.8%) than in patients with NSRT (17.2%) but this did not achieve statistical significance (*p* = 0.10). That finding may reflect the small numbers in group 2, and our advice to patients remains the same, that any patient with a history of a retinal tear in the fellow eye needs to be aware of the risk of a delayed onset retinal break in their fellow eye.

Given that the clinical diagnosis of retinal tears at initial examination has been reported to be of 89% [72%–98%], some of the SRT may represent missed tears at the initial examination [22]. In our series, we excluded patients who presented with media opacity precluding visualization of fundus details. Media opacity can be a confounding factor when making the diagnosis of retinal tears, as reported by Karahan, et al. [23]

Schweitzer et al. [24], have observed that patients with multiple floaters or/and vitreous or retinal hemorrhage showed a 100% sensibility for delayed retinal tears. In our series, patients who developed vitreous hemorrhage or retinal detachment after the treatment of retinal tear/s diagnosed at the initial visit were more prone to develop SRT during follow-up.

Dayan et al. [20], observed that reduction in vision was the most important factor to determine a retinal tear, although floaters or flashes should be carefully follow up at least 6 months. In our study, as well as in those from Dayan et al. [20], Jaffe [9], and Schweitzer et al. [24], symptomatic clinical presentation was more frequently observed in both groups, especially multiple floaters (NSRT 23.8% vs. SRT 36.4%, *p* = 0.21).

Although Sharma et al. [13], mentioned an association between retinal tears and patients with prior history of intra and extracapsular cataract extraction, in our study no association was observed between retinal tears and pseudophakia (NSRT 9.7% vs. SRT 9.1%, *p* = 0.66), maybe because all patients were operated on by phacoemulsification.

It has been found that the most frequent localization for SRT is the superotemporal quadrant [13]. However, in our series inferotemporal quadrant was more prevalent for SRT, although no statistical significance was found when comparing with NSRT (ST: NSRT 45.4% vs. SRT 40.1%, *p* = 0.94; IT: NSRT 30.1% vs. SRT 44.4%, *p* = 0.3). It should be necessary to enlarge our series in SRT group in order to analyze that retinal tear localization should be consider as a risk factor to develop SRT in retinal tear/s found in PVD course. When initial retinal tear localization was compared in SRT group (1-SRT) vs. second retinal tear event in the same group (2-SRT) there was no statistical differences.

Sharma et al. [13], observed in their series that 28% of patients with SRT developed a retinal detachment during following-up. In our series this complication was observed in 36.4% of patients (*p* = 0.001).

Patients with retinal detachment at initial presentation were not statistically significant in neither group, SRT 4.55% (1) and NSRT 1.3% (2) (p 0.27). Nevertheless, the risk to develop a retinal detachment during follow-up was higher in SRT than in NSRT, with 36.4% (8) and 9.74% (15), respectively (*p* = 0.0005).

Myopia was the independent most important related risk factor for SRT. It was found in 86.4% of eyes with SRT, and myopic eyes had a 4.5-fold risk for SRT when compared with non-myopic eyes.

Initial visual acuity was not a risk marker for SRT (*p* = 0.26). Logistic regression showed that reduced visual acuity at initial presentation was associated with history of retinal detachment or a retinal tear in the fellow eye (*p* = 0.03). Reduced visual acuity at initial presentation was inversely correlated with axial length (*p* = 0.01).

Sometimes the posterior vitreous cortex separation may be initially localized to the area of the initial retinal tear/s, and with PVD evolution and eventual traction over other focal anomalous vitreoretinal adhesions new retinal tears may occur in the following days or weeks.

Some authors have reported that the majority of retinal tears occurring in the context of a symptomatic PVD are observed within 2 to 6 weeks following the initial symptoms of PVD [4, 9, 20, 24]. In our study, 81.8% of the last diagnosed SRT occurred during a four-month period that followed the initially diagnosed retinal tear, 90.9% occurred throughout the first-year follow-up period, and only two cases were observed beyond 1 year of follow-up. This has also been found by Sharma et al. [13], considering that 1 year of follow-up should be largely enough to diagnose almost all SRT in the course of PVD.

The retrospective nature and the small number of cases in the SRT group are two important limitations of our study.

## Conclusions

Multiple retinal tears may be diagnosed simultaneously or subsequently in the evolution of a PVD, although SRT are less frequent than NSRT. SRT are most frequently observed in myopic eyes, and are commonly symptomatic. In almost two thirds of cases, SRT occur within 60 days after the initial finding of a retinal tear. The occurrence of a retinal detachment is more probably observed among patients with symptomatic PVD. PVD needs a close follow-up for at least 4 months after initial symptoms and/or diagnosis, especially in myopic patients and in those with a history of retinal detachment or retinal tear in the fellow eye.

## Abbreviations

CI: Confidence interval
NSRT: Non-subsequent retina tear
OR: Odd ratio
PVD: Posterior vitreous detachment
SRT: Subsequent retinal tear
